# Supplementary material for: Inferring fine-scale rates of mutation and recombination in the coppery titi monkey (Plecturocebus cupreus)
Source: Heredity (Edinb). 2026 Jul 21;135(7):476–85. doi: 10.1038/s41437-026-00853-6 (PMC13451275; doi:10.1038/s41437-026-00853-6)
Supplement: Supplementary file 1 [file 41437_2026_853_MOESM1_ESM.pdf]

chromosome	length	# SNPs	Ts/Tv	# invariable sites
1	235,103,535	644,311	2.64	148,205,777
2	158,738,102	434,111	2.70	98,497,389
3	165,937,890	467,368	2.56	102,000,154
4	151,623,469	383,446	2.65	94,167,465
5	150,993,727	430,053	2.60	94,005,171
6	123,740,199	345,869	2.69	76,193,309
7	135,512,294	367,789	2.52	82,843,393
8	116,834,941	346,007	2.47	72,431,464
9	101,234,152	273,083	2.58	60,149,663
10	75,878,286	213,058	2.77	44,393,010
11	49,373,254	131,462	2.66	29,936,619
12	231,176,762	586,920	2.66	141,745,863
13	118,029,634	282,023	2.80	73,673,212
14	130,776,705	283,266	2.71	81,241,012
15	95,533,141	249,431	2.58	59,771,475
16	94,172,006	247,341	2.59	59,156,815
17	73,180,778	194,592	2.83	44,439,300
18	92,880,398	286,221	2.53	57,538,597
19	65,768,754	198,900	2.91	42,772,996
20	91,752,637	241,048	2.42	54,790,595
21	73,432,007	182,368	2.55	44,848,075
22	44,717,611	116,987	2.52	27,199,013
∑ or ∅	**2,576,390,282**	**6,905,654**	**2.63**	**1,590,000,367**

Supplementary Table S1. Population genomic data.

window size	accessibility threshold			mutation rates					
	min. accessible length (bp)	number of windows that meet threshold	proportion of windows that meet threshold	6 year generation time			9 year generation time		
				32 mya	33 mya	36 mya	32 mya	33 mya	36 mya
1 Mb	10,000	4,295	0.857	1.051E-08	1.019E-08	9.344E-09	1.577E-08	1.529E-08	1.402E-08
	25,000	4,163	0.830	1.049E-08	1.017E-08	9.327E-09	1.574E-08	1.526E-08	1.399E-08
	50,000	3,975	0.793	1.048E-08	1.016E-08	9.314E-09	1.572E-08	1.524E-08	1.397E-08
	75,000	3,741	0.746	1.049E-08	1.017E-08	9.322E-09	1.573E-08	1.525E-08	1.398E-08
	100,000	3,428	0.684	1.050E-08	1.018E-08	9.336E-09	1.575E-08	1.528E-08	1.400E-08
	250,000	1,589	0.317	1.064E-08	1.032E-08	9.461E-09	1.597E-08	1.548E-08	1.419E-08
100 kb	1,000	37,164	0.923	1.045E-08	1.013E-08	9.290E-09	1.568E-08	1.520E-08	1.393E-08
	2,500	35,493	0.882	1.044E-08	1.012E-08	9.280E-09	1.566E-08	1.518E-08	1.392E-08
	5,000	33,155	0.824	1.045E-08	1.014E-08	9.292E-09	1.568E-08	1.520E-08	1.394E-08
	7,500	30,968	0.769	1.048E-08	1.016E-08	9.315E-09	1.572E-08	1.524E-08	1.397E-08
	10,000	28,894	0.718	1.051E-08	1.019E-08	9.339E-09	1.576E-08	1.528E-08	1.401E-08
	25,000	17,274	0.429	1.063E-08	1.031E-08	9.452E-09	1.595E-08	1.547E-08	1.418E-08
10 kb	100	254,635	0.977	1.052E-08	1.020E-08	9.351E-09	1.578E-08	1.530E-08	1.403E-08
	250	249,129	0.956	1.051E-08	1.020E-08	9.346E-09	1.577E-08	1.529E-08	1.402E-08
	500	241,325	0.926	1.051E-08	1.019E-08	9.343E-09	1.577E-08	1.529E-08	1.401E-08
	750	234,081	0.898	1.051E-08	1.020E-08	9.346E-09	1.577E-08	1.529E-08	1.402E-08
	1,000	226,709	0.870	1.052E-08	1.020E-08	9.349E-09	1.578E-08	1.530E-08	1.402E-08
	2,500	174,243	0.668	1.056E-08	1.024E-08	9.384E-09	1.583E-08	1.535E-08	1.408E-08
1 kb	10	1,933,294	0.971	1.074E-08	1.042E-08	9.548E-09	1.611E-08	1.562E-08	1.432E-08
	25	1,890,310	0.949	1.068E-08	1.035E-08	9.490E-09	1.602E-08	1.553E-08	1.424E-08
	50	1,838,381	0.923	1.063E-08	1.031E-08	9.450E-09	1.595E-08	1.546E-08	1.417E-08
	75	1,792,451	0.900	1.061E-08	1.028E-08	9.428E-09	1.591E-08	1.543E-08	1.414E-08
	100	1,748,751	0.878	1.059E-08	1.027E-08	9.414E-09	1.589E-08	1.541E-08	1.412E-08
	250	1,470,597	0.738	1.053E-08	1.022E-08	9.364E-09	1.580E-08	1.532E-08	1.405E-08

Supplementary Table S2. Mean mutation rates for a variety of window sizes, accessibility thresholds, generation times, and *P. cupreus*–*H. sapiens* divergence times.

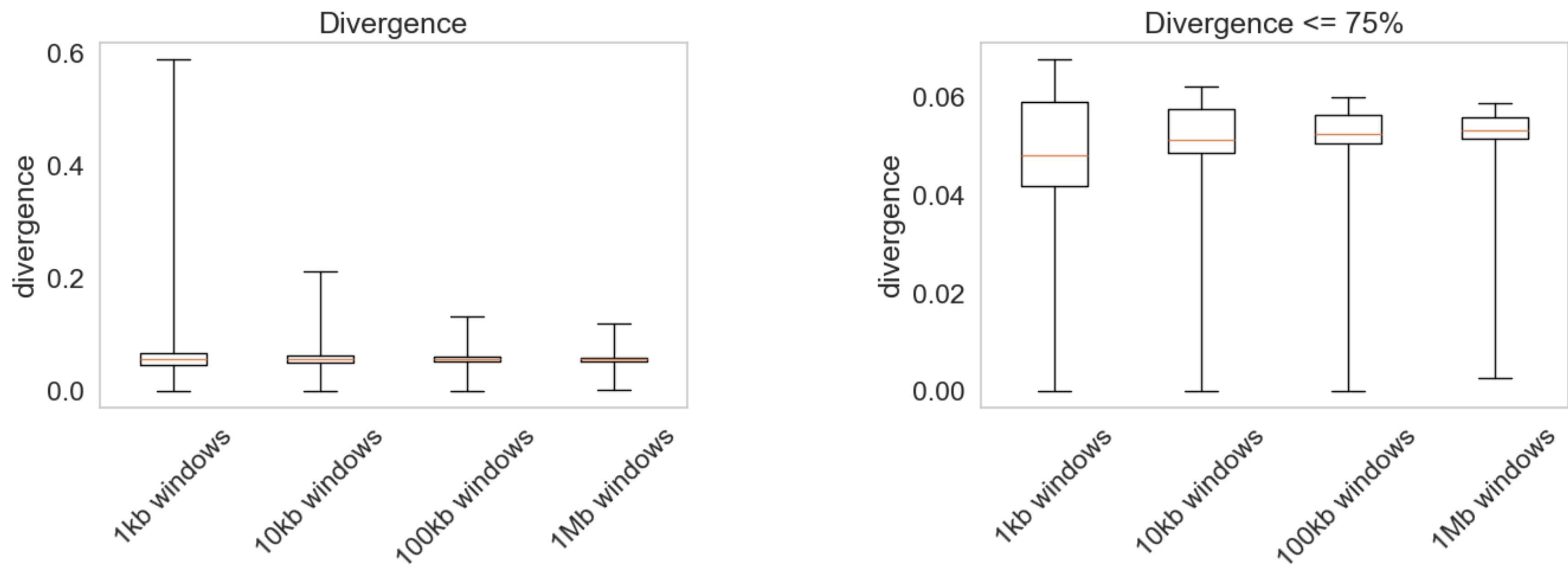

Supplementary Figure S1. Distributions of neutral divergence between *P. cupreus* and *H. sapiens* for all 1 kb, 10 kb, 100 kb and 1 Mb genomic windows (left) and for windows in the lower three quartiles (right). Orange bars represent the median neutral divergence, boxes represent the first and third quartiles, and whiskers represent the minimum and maximum values.

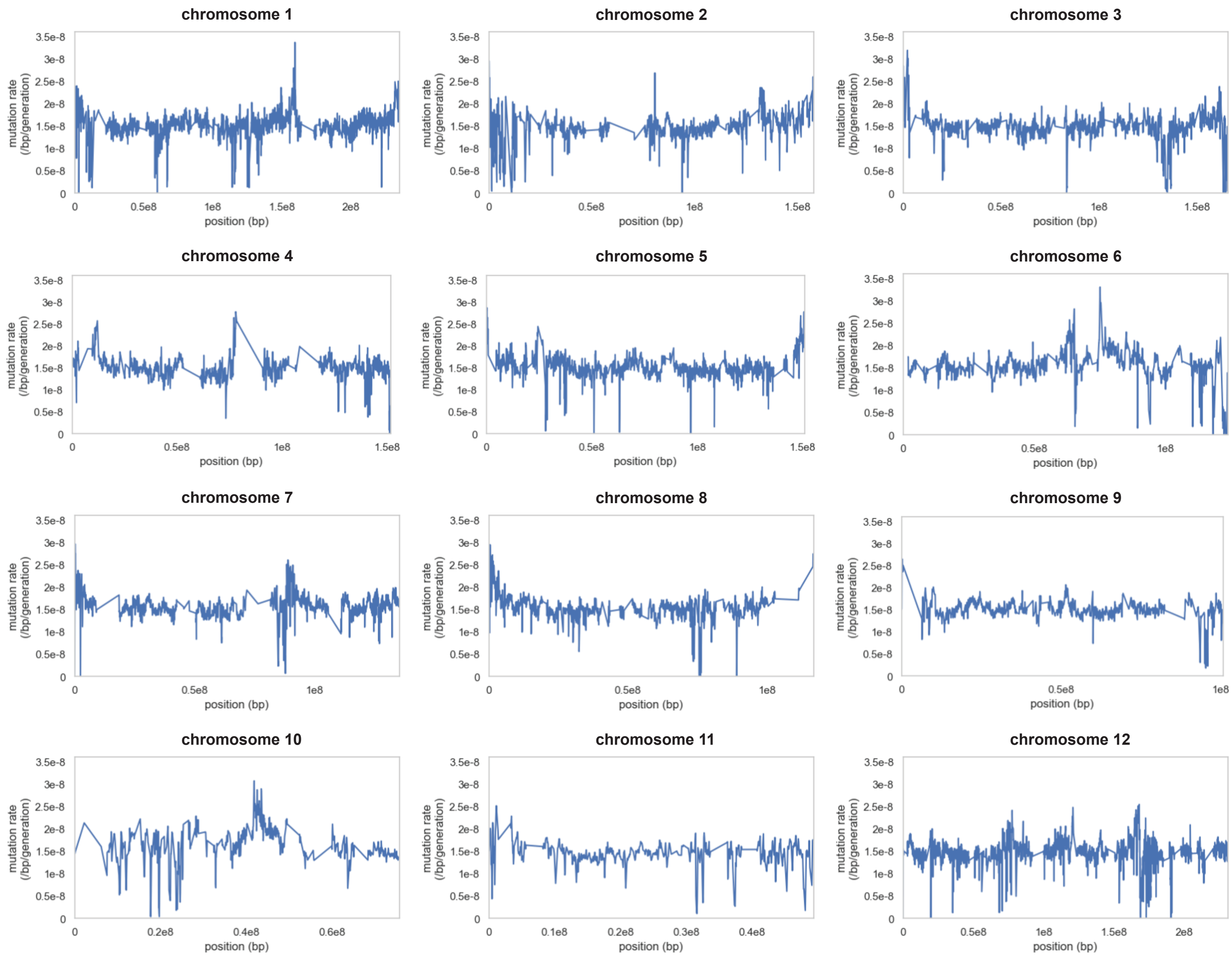
chromosome 1
chromosome 2
chromosome 3
chromosome 4
chromosome 5
chromosome 6
chromosome 7
chromosome 8
chromosome 9
chromosome 10
chromosome 11
chromosome 12
mutation rate (/bp/generation)
position (bp)
0
0.5e-8
1e-8
1.5e-8
2e-8
2.5e-8
3e-8
3.5e-8
0.5e8
1e8
1.5e8
2e8
0.1e8
0.2e8
0.3e8
0.4e8
0.6e8

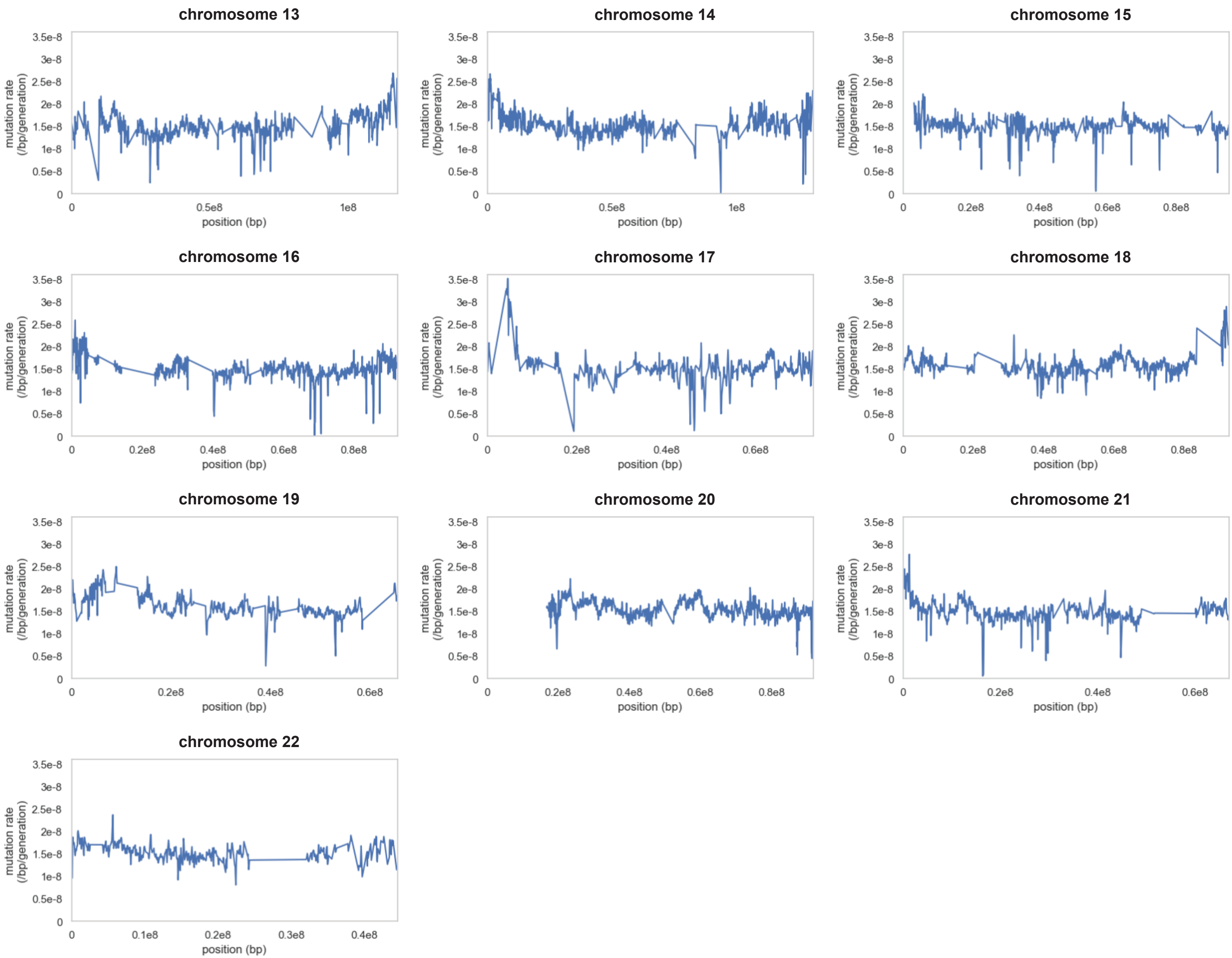

Supplementary Figure S2. Per-site per-generation neutral mutation rates across the autosomes for genomic windows of size 100 kb, with a 50 kb step size, assuming a *P. cupreus–H. sapiens* divergence time of 33 mya and a generation time of 9 years.

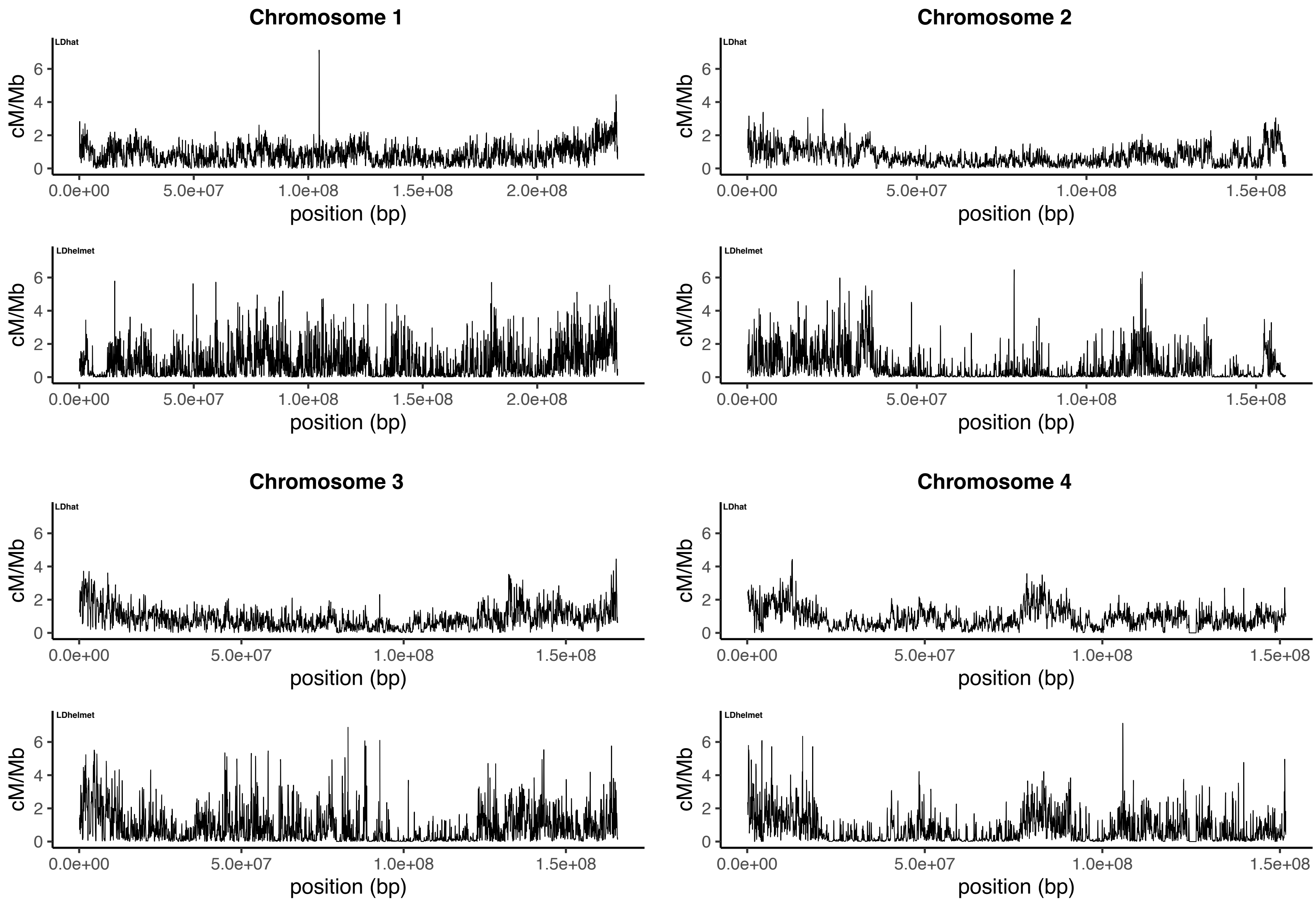

Chromosome 1
Chromosome 2
Chromosome 3
Chromosome 4
LDhat
LDhelmet
cM/Mb
position (bp)
0
2
4
6
0.0e+00
5.0e+07
1.0e+08
1.5e+08
2.0e+08

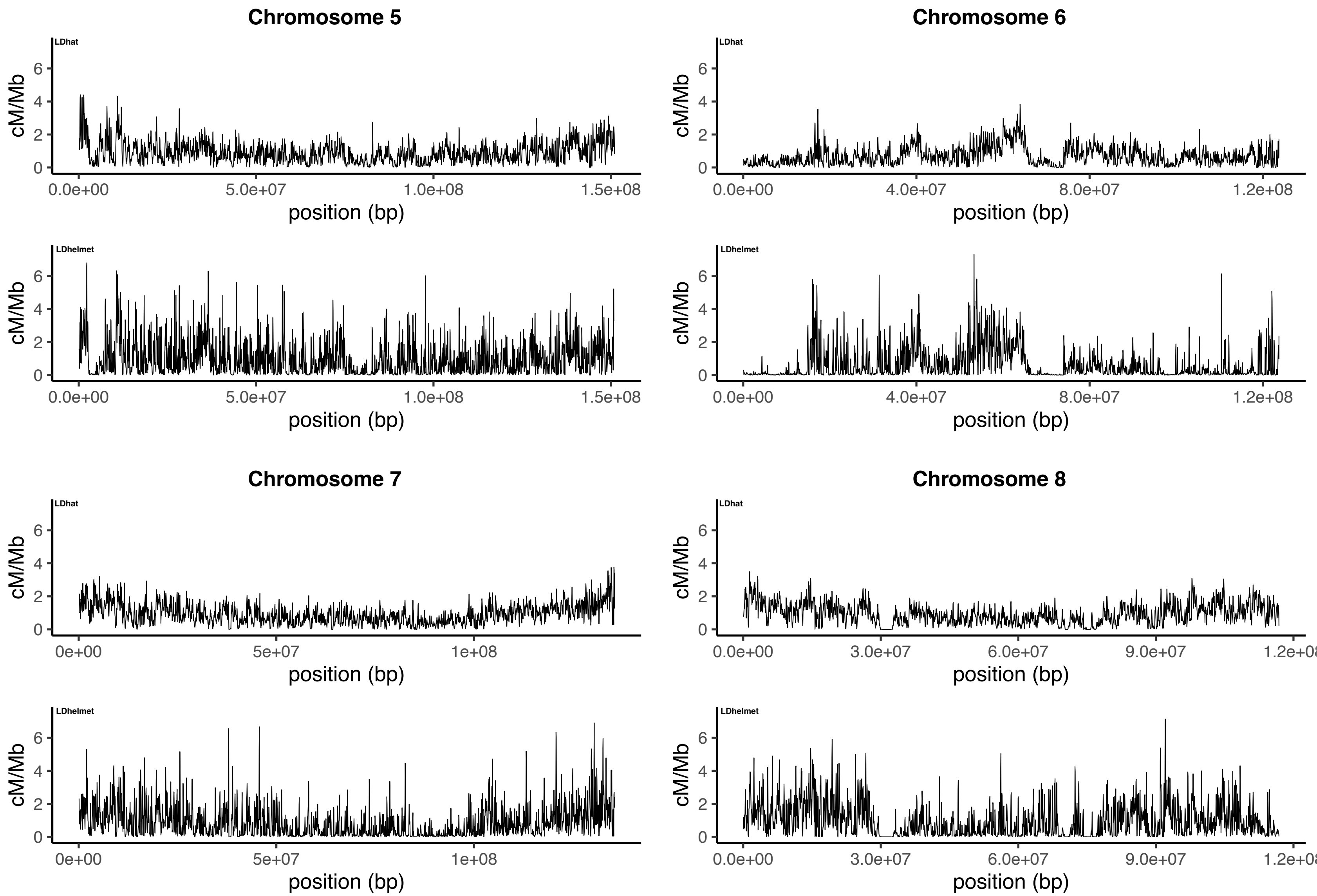
Chromosome 5
LDhat
cM/Mb
0
2
4
6
0.0e+00
5.0e+07
1.0e+08
1.5e+08
position (bp)
LDhelmet
cM/Mb
0
2
4
6
0.0e+00
5.0e+07
1.0e+08
1.5e+08
position (bp)
Chromosome 6
LDhat
cM/Mb
0
2
4
6
0.0e+00
4.0e+07
8.0e+07
1.2e+08
position (bp)
LDhelmet
cM/Mb
0
2
4
6
0.0e+00
4.0e+07
8.0e+07
1.2e+08
position (bp)
Chromosome 7
LDhat
cM/Mb
0
2
4
6
0e+00
5e+07
1e+08
position (bp)
LDhelmet
cM/Mb
0
2
4
6
0e+00
5e+07
1e+08
position (bp)
Chromosome 8
LDhat
cM/Mb
0
2
4
6
0.0e+00
3.0e+07
6.0e+07
9.0e+07
position (bp)
LDhelmet
cM/Mb
0
2
4
6
0.0e+00
3.0e+07
6.0e+07
9.0e+07
position (bp)

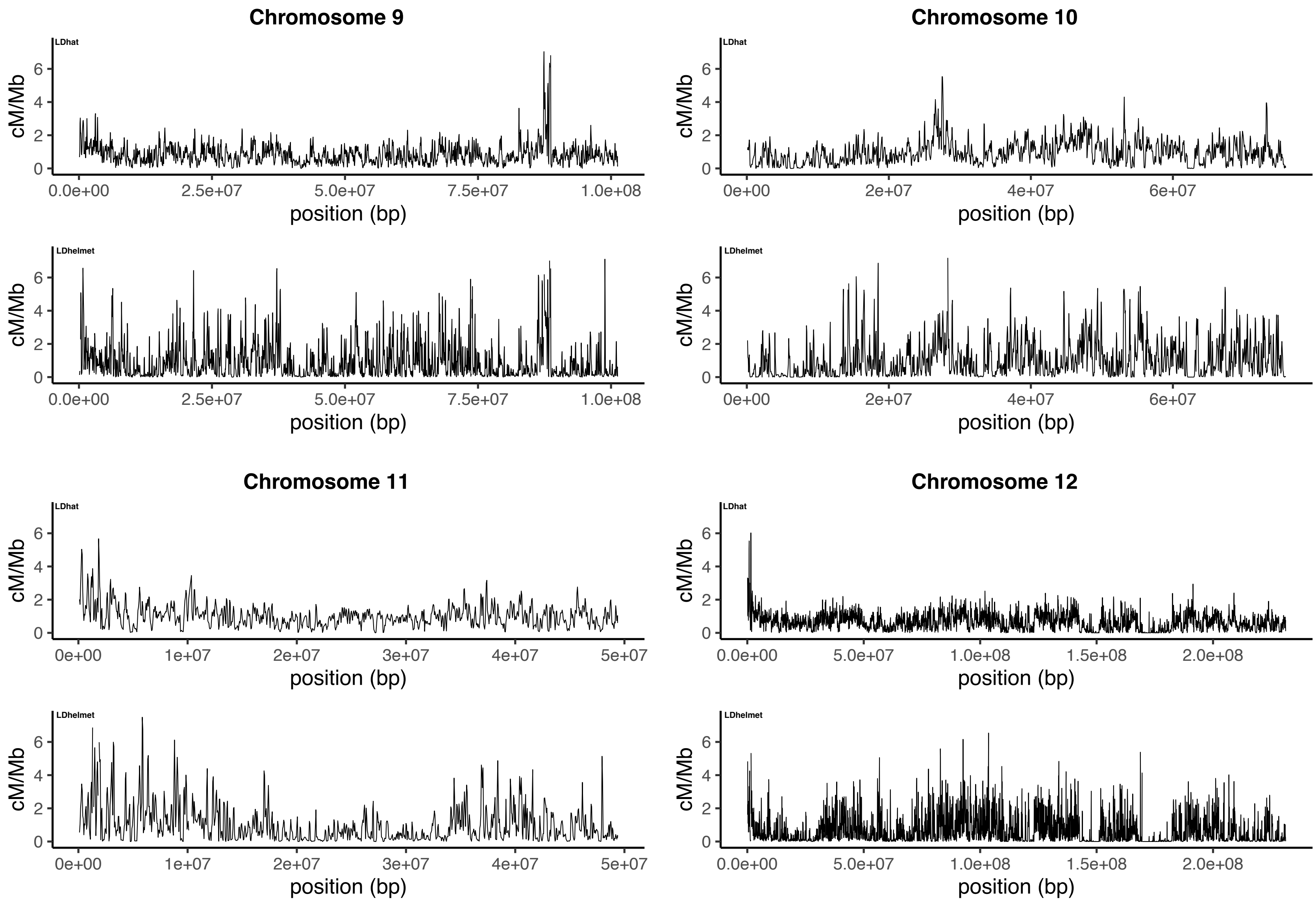

Chromosome 9
LDhat
cM/Mb
0
2
4
6
0.0e+00
2.5e+07
5.0e+07
7.5e+07
1.0e+08
position (bp)
LDhelmet
cM/Mb
position (bp)
Chromosome 10
LDhat
cM/Mb
0e+00
2e+07
4e+07
6e+07
position (bp)
LDhelmet
cM/Mb
position (bp)
Chromosome 11
LDhat
cM/Mb
0e+00
1e+07
2e+07
3e+07
4e+07
5e+07
position (bp)
LDhelmet
cM/Mb
position (bp)
Chromosome 12
LDhat
cM/Mb
0.0e+00
5.0e+07
1.0e+08
1.5e+08
2.0e+08
position (bp)
LDhelmet
cM/Mb
position (bp)

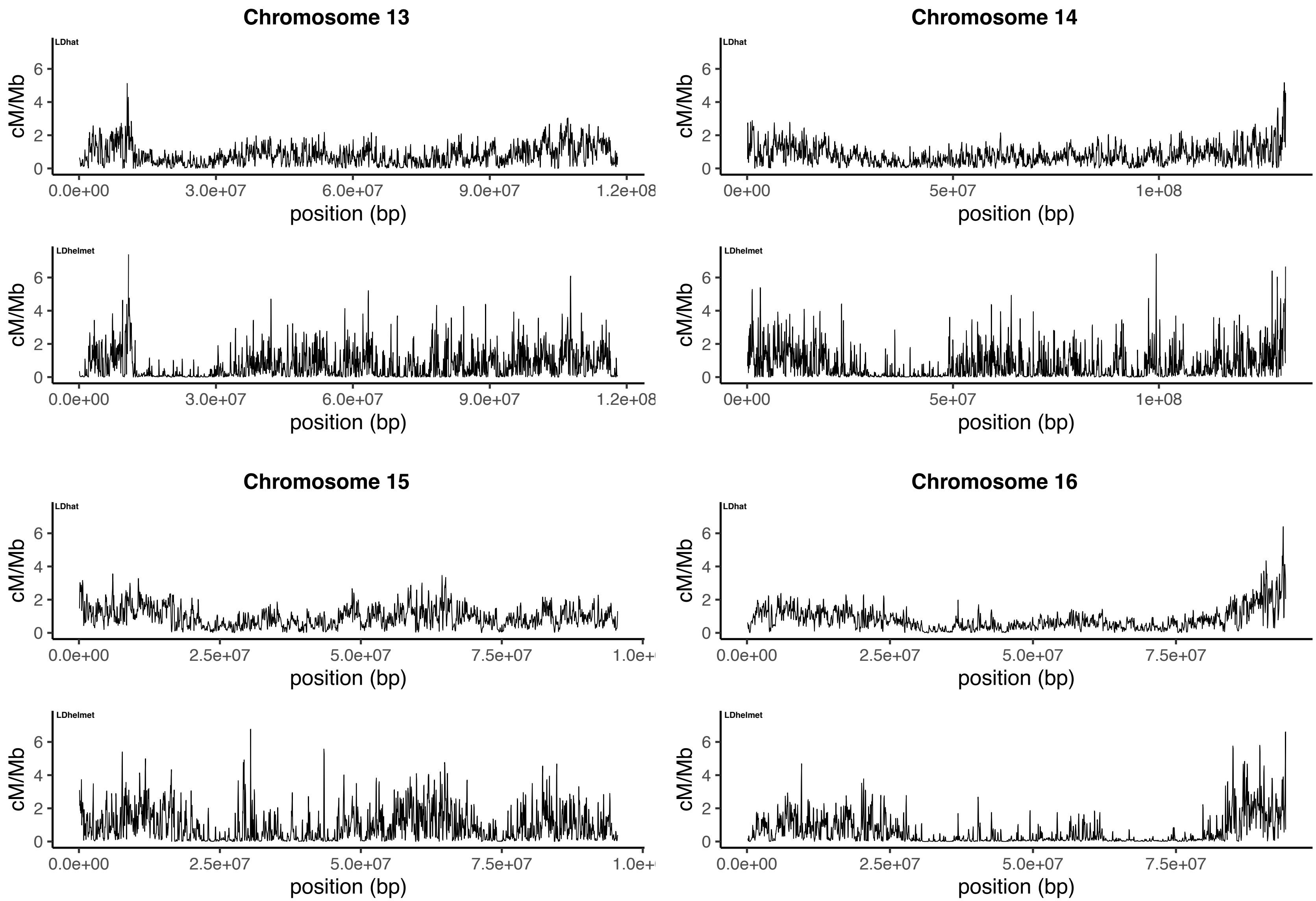
Chromosome 13
LDhat
cM/Mb
0
2
4
6
0.0e+00
3.0e+07
6.0e+07
9.0e+07
1.2e+08
position (bp)
LDhelmet
Chromosome 14
0e+00
5e+07
1e+08
Chromosome 15
2.5e+07
5.0e+07
7.5e+07
1.0e+
Chromosome 16
0.0e+00

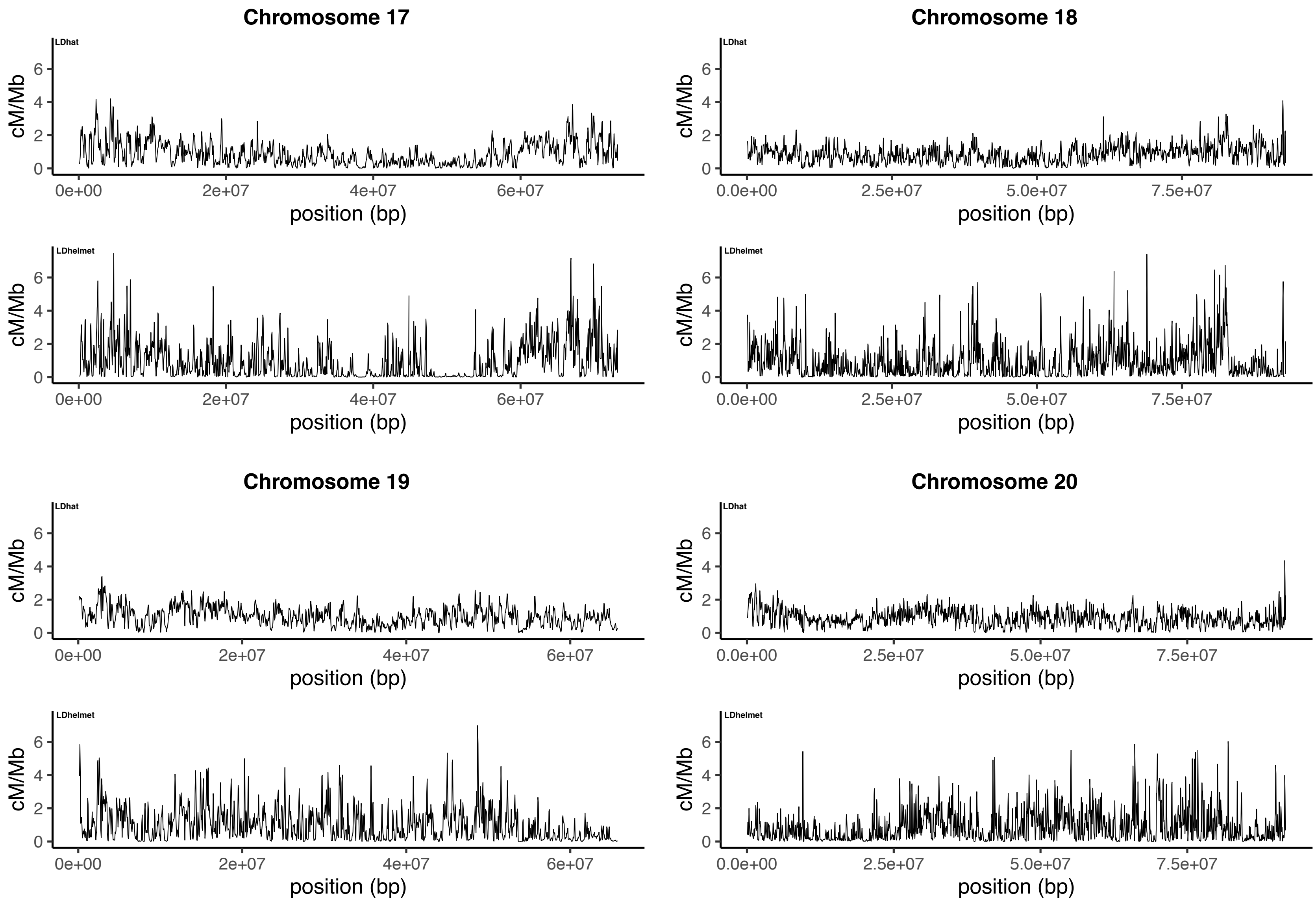
Chromosome 17
Chromosome 18
Chromosome 19
Chromosome 20
LDhat
LDhelmet
cM/Mb
position (bp)
0e+00
2e+07
4e+07
6e+07
0.0e+00
2.5e+07
5.0e+07
7.5e+07

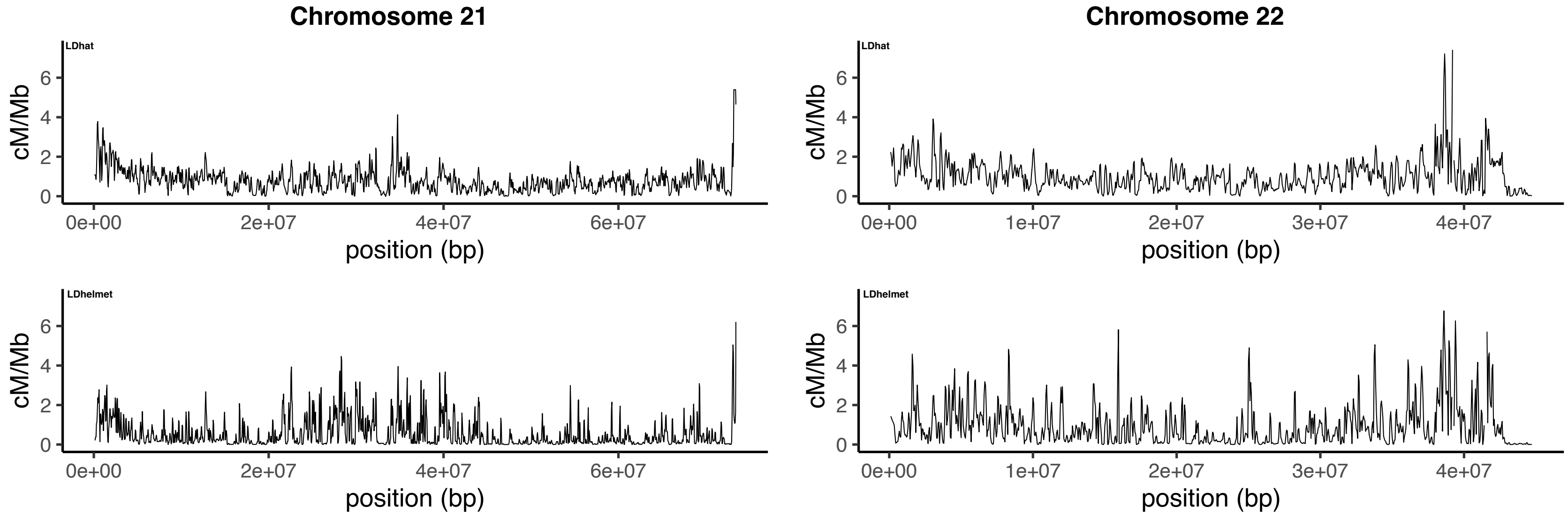

Supplementary Figure S3. Recombination rates inferred using LDhat (top) and LDhelmet (bottom) across the autosomes for genomic windows of size 1 Mb, with a 500 kb step size. Results for LDhat and LDhelmet are shown for a block penalty of 5.

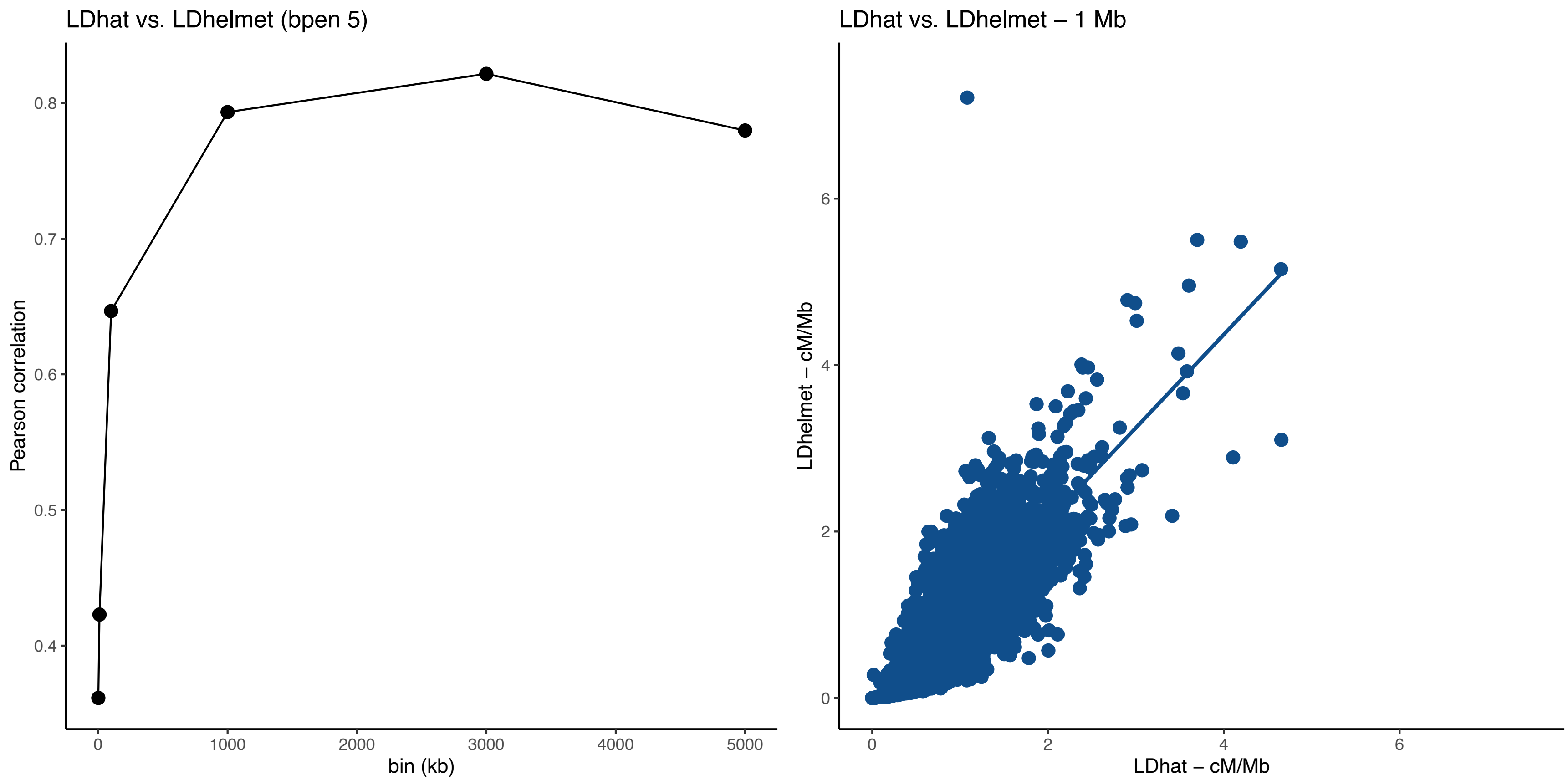

Supplementary Figure S4. Left panel: Pearson correlation between LDhat and LDhelmet recombination rate estimates calculated across non-overlapping genomic window of increasing size. **Right panel:** Pearson correlation between LDhat and LDhelmet recombination rate estimates at the 1 Mb scale. Results for LDhat and LDhelmet are shown for a block penalty of 5.
